# Maternal and fetal factors influencing fetal fraction: A retrospective analysis of 153,306 pregnant women undergoing noninvasive prenatal screening

**DOI:** 10.3389/fped.2023.1066178

**Published:** 2023-04-11

**Authors:** Cechuan Deng, Jianlong Liu, Sha Liu, Hongqian Liu, Ting Bai, Xiaosha Jing, Tianyu Xia, Yunyun Liu, Jing Cheng, Xiang Wei, Lingling Xing, Yuan Luo, Quanfang Zhou, Qian Zhu, Shanling Liu

**Affiliations:** ^1^ Prenatal Diagnostic Center, Department of Medical Genetics, West China Second University Hospital, Sichuan University, Chengdu, China; ^2^ Key Laboratory of Birth Defects and Related Diseases of Women and Children, Ministry of Education, Sichuan University, Chengdu, China

**Keywords:** cell-free DNA, noninvasive prenatal screening, fetal fraction, aneuploidy, genetic counseling

## Abstract

**Background:**

Genetic factors are important causes of birth defects. Noninvasive prenatal screening (NIPS) is widely used for prenatal screening of trisomy 21, trisomy 18, and trisomy 13, which are the three most common fetal aneuploidies. Fetal fraction refers to the proportion of cell-free fetal DNA in maternal plasma, which can influence the accuracy of NIPS. Elucidating the factors that influence fetal fraction can provide guidance for the interpretation of NIPS results and genetic counseling. However, there is currently no broad consensus on the known factors that influence fetal fraction.

**Objective:**

The study aimed to explore the maternal and fetal factors influencing fetal fraction.

**Methods:**

A total of 153,306 singleton pregnant women who underwent NIPS were included. Data on gestational age; maternal age; body mass index (BMI); z-scores for chromosomes 21, 18, and 13; and fetal fraction in NIPS were collected from the study population, and the relationships between fetal fraction and these factors were examined. The relationship between fetal fraction and different fetal trisomy types was also analyzed.

**Results:**

The results showed that the median gestational age, maternal age, and BMI of the pregnant women were 18 (16, 20) weeks, 29 (25, 32) years, and 22.19 (20.40, 24.24) kg/m^2^, respectively. The median fetal fraction was 11.62 (8.96, 14.7)%. Fetal fraction increased with gestational age and decreased with maternal age and BMI (*P *< 0.001). Fetal fraction of fetuses with trisomies 21, 18, and 13 was similar to that of the NIPS-negative group. The z-scores of pregnant women with trisomy 21 and 18 fetuses were positively correlated with fetal fraction, but not with that of the trisomy 13 cases.

**Conclusions:**

The factors that influence fetal fraction need to be taken into consideration before NIPS for quality control and after NIPS for result interpretation.

## Introduction

Cell-free fetal DNA (cffDNA) in maternal plasma, which is mainly derived from placental trophoblast cells, was first reported in 1997. cffDNA could be detected at 4 weeks of gestation, and its content increases and remains stable after 8 weeks of gestation (1). cffDNA in maternal plasma could be analyzed to screen for fetal aneuploidies; these methods are collectively called noninvasive prenatal screening (NIPS) (2). The detection rates of trisomy 21 (T21), trisomy 18 (T18), and trisomy 13 (T13) by NIPS, which are the three most common fetal aneuploidies, are 99%, 96%, and 91%, respectively, and the overall false-positive rate is less than 1% (2, 3).

Fetal fraction refers to the proportion of cffDNA in maternal plasma, and is approximately 10%–15% between 10 and 20 weeks of pregnancy (4, 5). However, it is impossible to separate the fetal and maternal cell-free DNA (cfDNA) completely during the NIPS process; therefore, tests involving cffDNA are affected by maternal cfDNA. Fetal fraction can influence the accuracy of NIPS; thus, understanding the factors affecting fetal fraction is crucial when interpreting NIPS results (6). Several research found that fetal fraction was affected by a variety of factors, such as placental size and function, maternal weight, and gestational age; fetal fraction is positively associated with gestational age (7) and negatively associated with maternal body mass index (BMI) (8, 9). In fact, no broad consensus about the known factors has been reached, and the sample sizes of previous studies were too small to obtain reliable results. Thus, a large amount of clinical sample data were retrospectively analyzed in this research to explore the influence of maternal age, maternal BMI, gestational age, and fetal trisomy on fetal fraction and determine the relationship between z-scores of chromosomes and fetal fraction in NIPS. The findings of this study can enhance the understanding and interpretation of NIPS results and further support its rational use.

## Materials and methods

### Subjects

The study enrolled 153,306 singleton pregnant women who underwent NIPS and successfully obtained fetal fraction and test results at the West China Second Hospital of Sichuan University from May 2015 to June 2020. Women with multiple pregnancies and those who had failed test results were excluded. Data on maternal age, gestational age, and BMI (i.e., calculated using height and body weight) of the pregnant women were collected. The pregnant women were divided into groups according to previous studies (10, 11) and the World Health Organization obesity classification system: (1) gestational age (weeks): group 1, 12–16; group 2, 17–20; group 3, 21–24; and group 4, ≥25; (2) maternal age (years): group 1, <25; group 2, 25–29; group 3, 30–34; group 4, 35–39; and group 5, ≥40; (3) BMI (kg/m^2^): group 1, <18.5; group 2, 18.5–<25; group 3, 25–<30; group 4, 30–<35; group 5, 35–<40; group 6, ≥40. Due to the small size of the groups with BMI ≥ 30 kg/m^2^, the results may be biased; therefore, the last three groups were combined into one group and the study population was re-divided into four BMI groups: (4) BMI (kg/m^2^): group 1, <18.5; group 2, 18.5–<25; group 3, 25–<30; and group 4, ≥30.

### Noninvasive prenatal screening

NIPS was performed by collecting 8–10 ml of peripheral blood from the expectant mother. Extraction of cfDNA, library construction, and massive parallel sequencing were carried out as described in a previous study (12). Fetal fraction of male and female fetuses were estimated based on the Y chromosome-based method (13) and distribution of plasma cfDNA fragment length, respectively (14). We set 4% as the lowest limit of fetal fraction for obtaining accurate NIPS results (15, 16). Z-score is the standard difference between the case data and mean of the reference dataset, which represents the statistical deviation of the count and ploidy status of a chromosome (17). *Z* ≥ 3 indicates that the chromosome is at a high risk of trisomy, while-3 < *z* < 3 indicates that the chromosome is at a low risk of trisomy.

### Invasive prenatal diagnosis

If the NIPS results indicated T21, T18, or T13, the pregnant women could voluntarily choose to proceed with invasive prenatal diagnosis after receiving genetic counseling. Invasive prenatal diagnosis methods included villus sampling or amniocentesis, and detection programs included diagnostic cytogenetic testing (18, 19), low-pass genome sequencing (20), or chromosomal microarray analysis (21).

### Statistical analysis

Kolmogorov–Smirnov test and Shapiro–Wilk test were applied to test whether the continuous variables were in normal distribution. Demographic data were expressed as medians and interquartile ranges. Then we performed Kruskal–Wallis *H* test for comparison of the differences among the variable levels, followed by the SNK-q test for the multiple comparisons. The association between gestational age, maternal age, BMI, trisomies, and fetal fraction was calculated by the Jonckheere–Terpstra trend test. The relationships between z-scores of chromosomes 21, 18, and 13 and fetal fraction were presented as scatter plots. Correlation analysis was performed using Spearman rank correlation coefficient. SAS software programs (SAS 9.4; SAS Institute, Inc.) was used for data analyses, and *P* < 0.05 was considered significant.

## Results

### Basic characteristics of the pregnant women

The gestational age, maternal age, BMI, and fetal fraction of 153,306 singleton expectant mother who chose NIPS at the West China Second Hospital of Sichuan University from May 2015 to June 2020 are presented in Table 1. The median gestational and maternal ages of the pregnant women were 18 weeks (range 16–20) and 29 years (range 25–32), respectively. The median maternal BMI was 22.19 kg/m^2^ (range 20.40–24.24) and the median fetal fraction of all samples was 11.62% (range 8.96–14.70).

**Table 1:** Basic characteristics and fetal fraction of the study population.

Characteristic	*n*	Median (Q1, Q3)
Gestational age (weeks)	153,306	18 (16, 20)
Maternal age (years)	153,306	29 (25, 32)
Maternal BMI (kg/m^2^)	153,306	22.19 (20.40, 24.24)
Fetal fraction (%)	153,306	11.62 (8.96, 14.70)

### The influence of gestational age on fetal fraction

**Figure 1:**
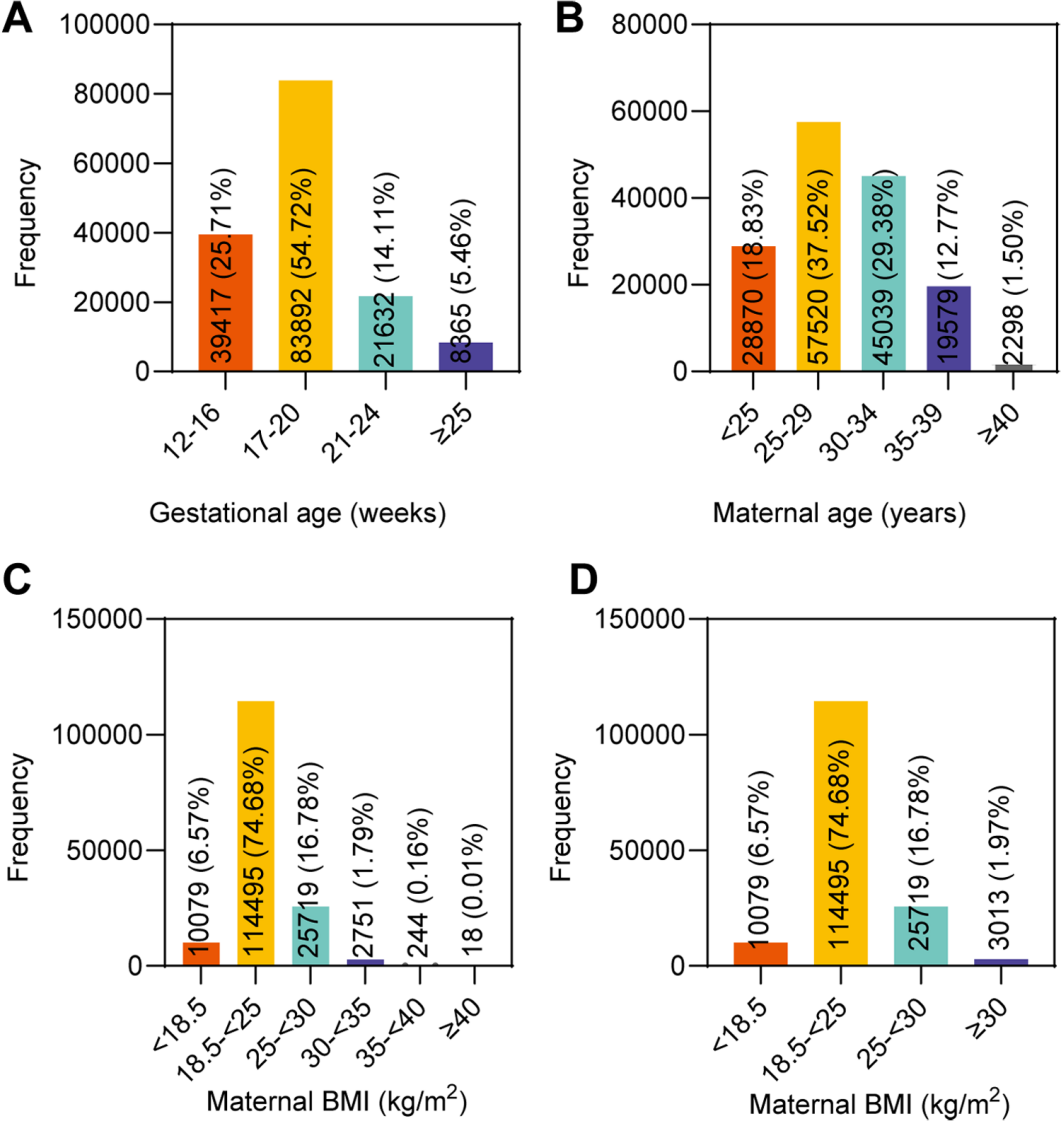
Distribution of maternal and fetal characteristics of the 153,306 singleton pregnant women in the study. (**A**) Gestational age. (**B**) Maternal age. (**C**) Maternal BMI in six groups. (**D**) Maternal BMI in four groups.

**Figure 2:**
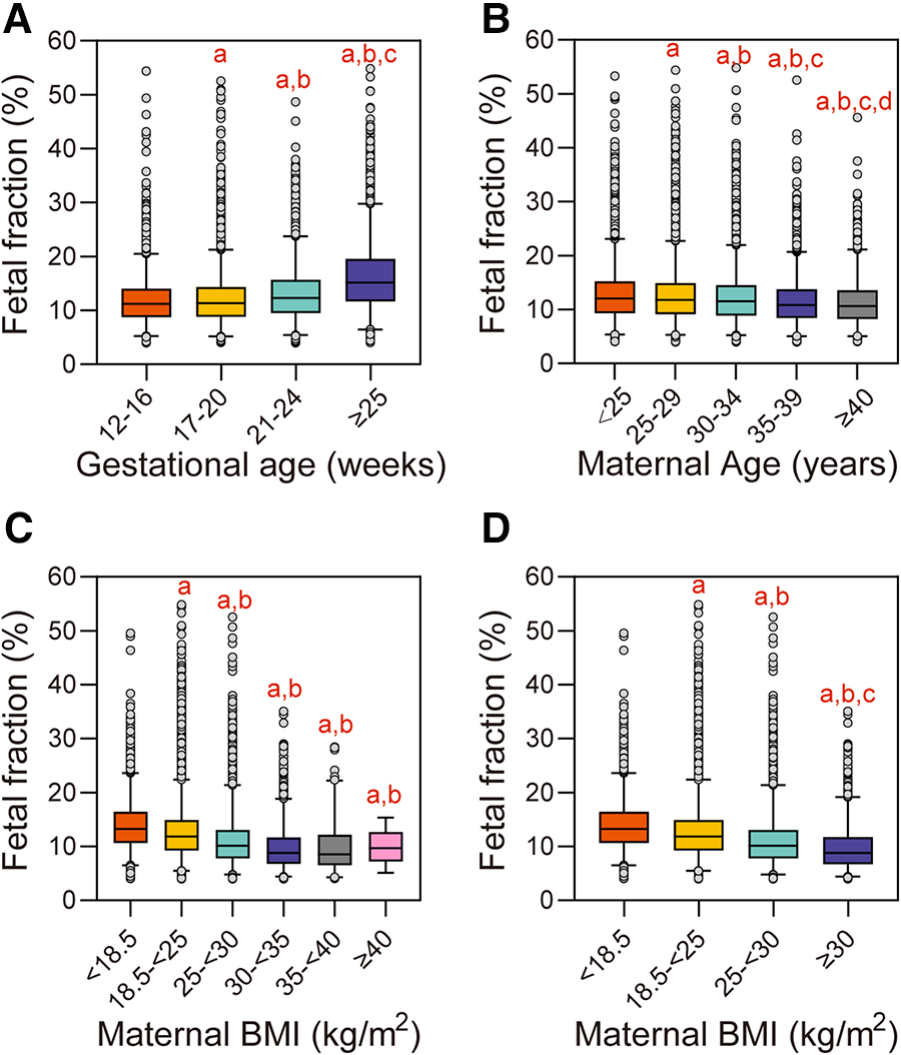
Influence of maternal and fetal characteristics on fetal fraction. (**A**) Gestational age. ^a^Reference group is 12–16 weeks, *P* < 0.05. ^b^Reference group is 17–20 weeks, *P* < 0.05. ^c^Reference group is 21–24 weeks, *P* < 0.05. (**B**) Maternal age. ^a^Reference group is <25 years, *P* < 0.05. ^b^Reference group is 25–29 years, *P* < 0.05. ^c^Reference group is 30–34 years, *P* < 0.05. ^d^Reference group is 35–39 years, *P* < 0.05. (**C**) Maternal BMI in the six groups. ^a^Reference group is <18.5 kg/m^2^, *P* < 0.05. ^b^Reference group is 18–<25 kg/m^2^, *P* < 0.05. (**D**) Maternal BMI in the four groups. ^a^Reference group is <18.5 kg/m^2^, *P* < 0.05. ^b^Reference group is 18–<25 kg/m^2^, *P* < 0.05. ^c^Reference group is 25–<30 kg/m^2^, *P* < 0.05.

Of the 153,306 pregnant women, 39,417 (25.71%) were assigned to the 12–16 gestational weeks group, 83,892 (54.72%) to the 17–20 gestational weeks group, 21,632 (14.11%) to the 21–24 gestational weeks group, and the remaining 8,365 (5.46%) pregnant women were in the ≥25 gestational weeks group (Figure 1A). The median fetal fraction in the 12–16 weeks group was 11.25% (8.74, 14.05). The fetal fraction of the 17–20 weeks, 21–24 weeks, and ≥25 weeks groups were higher compared to that of the 12–16 weeks group [11.37% (8.79, 14.35), 12.32% (9.49, 15.69), and 15.15% (11.66, 19.53), respectively, *P *< 0.05]. The comparison also showed that fetal fraction increased gradually from the 17–20 weeks group to the ≥25 weeks group (*P *< 0.05). Moreover, fetal fraction generally tended to increase with increasing gestational age (Jonckheere–Terpstra test, *P *< 0.001), and the increasing rate of fetal fraction was different at each stage (Figure 2A).

### The influence of maternal age on fetal fraction

When grouped by maternal age, 28,870 (18.83%) pregnant women were assigned to the <25 years group, 57,520 (37.52%) to the 25–29 years group, 45,039 (29.38%) to the 30–34 years group, 19,579 (12.77%) to the 35–39 years group, and the remaining 2,298 (1.50%) pregnant women were in the ≥40 years group (Figure 1B). The median fetal fraction in the <25 years group was 12.04% (9.3, 15.23). The fetal fraction in the 25–29 years, 30–34 years, 35–39 years, and ≥40 years groups were lower compared to that of the <25 years group [11.81% (9.11, 14.91), 11.51% (8.89, 14.54), 10.85% (8.4, 13.75), and 10.65% (8.23, 13.55), respectively, *P *< 0.05]. Compared with the 25–29 years, 30–34 years, and 35–39 years groups, the fetal fraction of the next groups decreased gradually, respectively (*P *< 0.05). Moreover, there was a general trend of decreasing fetal fraction with increasing maternal age (Jonckheere–Terpstra test, *P *< 0.001) (Figure 2B).

### The influence of maternal BMI on fetal fraction

The study population was first divided into six BMI groups; 10,079 (6.57%) pregnant women were assigned to the <18.5 kg/m^2^ group, 114,495 (74.68%) to the 18.5–<25 kg/m^2^ group, 25,719 (16.78%) to the 25–<30 kg/m^2^ group, 2,751 (1.79%) to the 30–<35 kg/m^2^ group, 244 (0.16%) to the 35–<40 kg/m^2^ group, and the remaining 18 (0.01%) to the ≥40 kg/m^2^ group (Figure 1C). The median fetal fraction of the <18.5 kg/m^2^ group was 13.28% (10.64, 16.42). The fetal fraction of the 18.5–<25 kg/m^2^ group, 25–<30 kg/m^2^ group, 30–<35 kg/m^2^ group, 35–<40 kg/m^2^ group, and ≥40 kg/m^2^ group were lower compared with that of the <18.5 kg/m^2^ group [11.87% (9.24, 14.89), 10.16% (7.81, 13.06), 8.82% (6.74, 11.67), and 9.72% (7.3, 12.17), respectively, *P *< 0.05]. In addition, the fetal fraction of the 25–<30 kg/m^2^, 30–<35 kg/m^2^ group, 35–40 kg/m^2^, and ≥40 kg/m^2^ groups were lower compared with that of the 18.5–<25 kg/m^2^ group (*P *< 0.05). Moreover, fetal fraction generally tended to decrease with increasing maternal BMI in the six groups (Jonckheere–Terpstra test, *P *< 0.001) (Figure 2C).

In order to avoid the bias due to the small size of samples with BMI greater than 30 kg/m^2^, and to further elucidate the relationship between fetal fraction and BMI, the three groups with BMI ≥ 30 kg/m^2^ were combined into one group; thus, there were four groups in the final analysis. A total of 3,013 (1.97%) pregnant women were in the ≥30 kg/m^2^ group (Figure 1D). The fetal fraction of the 18.5–<25 kg/m^2^, 25–<30 kg/m^2^, and ≥30 kg/m^2^ groups were lower compared with that of the <18.5 kg/m^2^ group [11.87% (9.24, 14.89), 10.16% 7.81, 13.06), and 8.81% (6.73, 11.7), respectively, *P *< 0.05]. The fetal fraction of the 25–<30 kg/m^2^ and ≥30 kg/m^2^ groups were lower (*P *< 0.05) compared with that of the 18.5–<25 kg/m^2^ group. In addition, compared with the 25–<30 kg/m^2^ group, the fetal fraction of the ≥30 kg/m^2^ group was lower (*P *< 0.05). Similarly, fetal fraction generally tended to decrease with increasing maternal BMI in the four groups (Jonckheere–Terpstra test, *P *< 0.001) (Figure 2D).

### The influence of fetal trisomy on fetal fraction

A total of 492 pregnant women were suspected to have target trisomy by NIPS. Of which, 87 refused invasive prenatal diagnosis and 405 (82.32%) chose invasive prenatal diagnosis. Of the 87 pregnant women who refused invasive prenatal diagnosis, 28 terminated the pregnancy due to subsequent ultrasound structural abnormalities, 2 terminated the pregnancy due to personal reasons, 28 terminated the pregnancy for unknown reasons, 8 had intrauterine fetal deaths, 6 chose to continue with the pregnancy, and 15 were lost to follow-up. These cases were not included in the analysis because confirmatory tests were not performed. Among the pregnant women selected for invasive prenatal diagnosis, one woman was suspected to be carrying fetuses positive for both T18 and T13 by NIPS, while another woman was suspected to be carrying fetuses positive for both T21 and T18. Confirmatory tests showed that 282 cases were true positives, of which the number of T21, T18 and T13 cases is 243, 31, and 8 respectively. Of the 152,935 NIPS-negative cases, seven false-negative cases were followed-up, including three T21 cases and four T18 cases. Moreover, there were 8 of the 153,306 cases with fetal fraction >50%. The median gestational age and maternal age of the pregnant women were 23 weeks (range 18–28) and 30 years (range 28–31), respectively. The median maternal BMI was 23.75 kg/m^2^ (range 23.15–25.99) and the median fetal fraction of all samples was 51.73% (range 50.71–53.56). The NIPS results of these samples with high fetal fraction all indicated negative, and no false negative was found in the follow-up.

**Figure 3:**
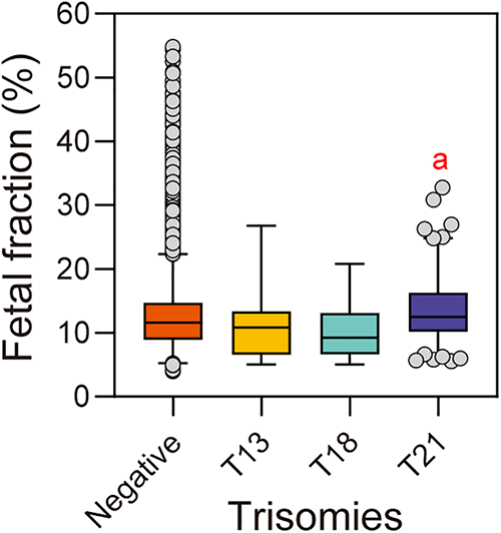
Fetal fraction of pregnant women with different types of trisomy fetuses. ^a^Reference group is T18, *P* < 0.05.

Figure 3 shows the fetal fraction of the pregnant women with different trisomy types. The median fetal fraction of the T21 group (*n *= 243) was 12.49% (10.19, 16.27), which was higher than that of the NIPS-negative group [*n *= 152,935, 11.62% (8.96, 14.7)]. The median fetal fraction of the T18 (*n *= 31) and T13 (*n *= 8) groups were 9.28% (6.67, 13.15) and 10.86% (7.54, 12.86), respectively, which were lower than that of the NIPS-negative group. However, the comparison between groups revealed differences in fetal fraction only between the T18 and T21 groups, whereas there was no difference between the other groups.

### The relationship between chromosome z-score and fetal fraction

**Figure 4:**
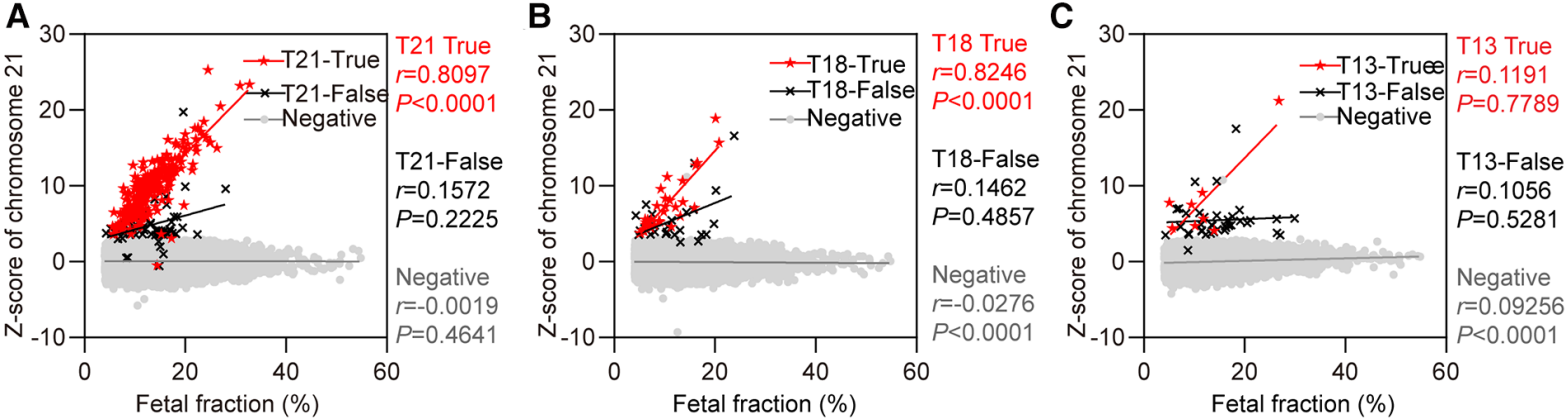
The relationship between z-score of chromosomes and fetal fraction. (**A**) Chromosome 21. Red solid five-pointed stars represent false-positive cases (*n *= 62). (**B**) Chromosome 18. Red solid five-pointed stars represent false-positive cases (*n *= 25). (**C**) Chromosome 13. Red solid five-pointed stars represent false-positive cases (*n *= 38).

Figure 4 showed the relationship between the chromosome z-scores and fetal fraction in NIPS. The correlation analysis results between z-score and fetal fraction are presented in Table 2. It was found that both z-score of chromosome 21 in the T21 true positive group and z-score of chromosome 18 in the T18 true positive group were positively correlated with fetal fraction (*r *= 0.8097, *P *< 0.0001; *r *= 0.8246, *P *< 0.0001), while no correlation was found in the T13 true positive group. In addition, z-score of chromosome 18 and z-score of chromosome 13 in negative group were negatively correlated and positively correlated with fetal fraction (*r *= − 0.0276, *P *< 0.0001; *r *= 0.09256, *P *< 0.0001). Moreover, the z-scores of the true-positive samples tended to be larger than those of the false-positive samples.

**Table 2:** Relationship between the z-scores of chromosomes and fetal fraction.

	Z-score of chromosome 21			Z-score of chromosome 18			Z-score of chromosome 13		
	*n*	*r*	*P*	*n*	*r*	*P*	*n*	*r*	*P*
Fetal fraction of false group	62	0.1572	0.2225	25	0.1462	0.4857	38	0.1056	0.5281
Fetal fraction of true group	243	0.8097	<0.0001*	31	0.8246	<0.0001*	8	0.1191	0.7789
Fetal fraction of negative group	152,812	−0.0019	0.4641	152,812	−0.0276	<0.0001*	152,812	0.09256	<0.0001*

*P < 0.05.

## Discussion

NIPS is widely used for prenatal screening of T21, T18, and T13, and its quality control is very important. Fetal fraction is a crucial quality control parameter of NIPS, and there are many factors influencing it. However, there are few research on large-scale samples. Therefore, we conducted a retrospective analysis on gestational age, maternal age, BMI, and fetal karyotype of 153,306 singleton expectant mother who chose NIPS in the West China Second Hospital of Sichuan University, to evaluate the effects of these factors on fetal fraction in large scale of population, and to discuss the relationship between fetal fraction and z-scores in NIPS.

Analysis of gestational age in this research found that fetal fraction was affected by gestational age, and fetal fraction was positively correlated with gestational age in general. Fetal fraction increased from 17–20 weeks and continued to rise from 21–24 weeks, which was similar to the result of one study (10) that showed fetal fraction accelerated from 19 to 23 weeks and another study (22) that showed fetal fraction increased greatly from 20 weeks. Moreover, the increasing rate differed at different stages of pregnancy. Taken together, the results indicated that gestational age is a factor influencing fetal fraction. The possible reason is that with the increase of gestational age, the placental volume increases gradually, and the apoptotic trophoblast cells also increase gradually. As a result, more cffDNA fragments are released into maternal plasma, resulting in an increase in fetal fraction, that is, fetal fraction increases with gestational age.

Furthermore, the study revealed that fetal fraction tends to decrease with an increase in maternal BMI. Moreover, the three groups with BMI ≥ 30 kg/m^2^ were combined into one group and re-analyzed. Our analysis revealed that the fetal fraction decreases with an increase in BMI, which was in line with previous results (10). In addition, our previous study analyzed test failure cases of NIPS, and the primary reason (79.44%) for test failure in 394/123,291 cases was fetal fraction <4%, other reasons included sequencing failure and DNA concentration higher than the quality control standard (12). The gestational age of the low fetal fraction group was 17.40 ± 2.27 weeks and the BMI was 25.66 ± 3.67 kg/m^2^, which were significantly different from those of the sequencing failure group and the DNA concentration outside of laboratory quality control group (all *P* < 0.05). This suggested that low gestational age and high BMI tend to lead to low fetal fraction, even NIPS detection failure, which is similar to previous studies (23). Maternal obesity is associated with increased blood volume, fat cell turnover, and interstitial cell apoptosis. With the increase of pregnant women's BMI, the increase of blood volume leads to the increase of the overall DNA content in the blood, thus diluting the cfDNA of fetal origin in peripheral blood, resulting in the decrease of fetal fraction. Another possible reason is that adipose tissue carries out active fat reconstruction through adipocyte necrosis or basal vascular tissue apoptosis in obese pregnant women. After adipocyte lysis, cfDNA is released into maternal blood. Therefore, maternal cfDNA in the plasma of an obese pregnant woman is not only derived from apoptotic hematopoietic cells, but also from apoptotic and necrotic cells of adipose and stromal vascular tissues (24). Thus, the decrease in the fetal fraction may be due to a dilution effect or relative decrease in cffDNA content due to the increase in maternal cfDNA (5, 9, 25–27). Based on these results, clinicians should consider the relationship between BMI and fetal fraction when choosing whether to perform NIPS in obese pregnant women. As gestational age increases, BMI also increases; therefore, these two factors may simultaneously affect the fetal fraction. When a NIPS test failure result is obtained, re-blood and re-test is a viable option. If the re-test still fails, genetic counseling, a thorough ultrasound evaluation, and prenatal diagnosis are recommended.

Most pregnant women in the study population were 25–35 years old, and more than one-third of the expectant mother were 25–30 years old, which was consistent with the main childbearing age of the Chinese population. Negative correlation between maternal age and fetal fraction was found in our research, and fetal fraction decreases with an increase in maternal age, which was in good agreement with previous results (10, 23). Several studies have shown that maternal age affects fetal fraction (28, 29), while others found no association between these two factors (30). A previous review also indicated that the relationship between maternal age and fetal fraction varied between studies (31). Fetal fraction decreased with maternal age, which may be related to pregnancy-induced complications, such as pregnancy-induced hypertension and antiphospholipid syndrome. In addition, prenatal diseases such as pre-eclampsia will increase maternally derived DNA greatly, and these cfDNA will be released into the maternal blood, thus diluting the percentage of fetal cfDNA and reducing the percentage of cffDNA. This may be one of the factors causing a low fetal fraction, which requires further investigation. Therefore, there is currently no broad consensus on the relationship between maternal age and fetal fraction, and further studies are required. Both the American College of Obstetricians and Gynecologists Committee on Genetics (32) and China's NIPS national norms suggest that pregnant women with an expected delivery age of ≥35 years should be cautious about undergoing NIPS. Although this is mainly due to the fact that the risk of fetal chromosomal abnormalities in older expectant mother is higher than that in younger expectant mother, the decrease of fetal fraction caused by the increase of maternal age may be avoided from the perspective of fetal fraction. However, it is not advisable to halt the usage of NIPS alone because of the possible decrease in fetal fraction caused by the increase in age, and a variety of factors should be considered comprehensively.

Our study also revealed that fetal fraction of the T21 group was larger than that of the NIPS-negative group, whereas those of the T18 and T13 groups were smaller. However, statistical analysis found that the fetal fraction of the NIPS-negative group was similar to that of the T21, T18, and T13 groups, and the difference was not statistically significant, which was similar to previous findings (5, 30). In addition, Palomaki et al. revealed significant differences between a higher fetal fraction for pregnant women with T21 fetuses and a lower fetal fraction for pregnant women with T18 or triploid fetuses compared with that with euploid fetuses (34). Contrarily, Suzumori et al. found that pregnant women with euploid (13.7%) and T21(13.6%) fetuses had comparable fetal fraction, and fetal fraction of pregnant women with T18 and T13 fetuses were 11.0% and 8.0%, respectively, which were significantly lower than that of euploid fetuses (25). These findings may be explained through the fetal growth restriction or smaller placental size in pregnant women with T18 and T13 fetuses, leading to a smaller fetal fraction than euploid fetuses. This may be the reason why detection of T21 by NIPS is more efficient but challenging for T18 and T13. Moreover, we compared true positives, false positives and negatives all together. In addition, false negatives are not listed separately because there are fewer cases, even fewer when grouped according to the categories of T21, T18 and T13. The number of false negatives was too small to be used for comparison between groups. Therefore, true positive, false positive and negative cases are compared. Our study found that the z-scores of chromosomes 21 and 18 were positively correlated with the fetal fraction in true positive groups, which matches well with previous research (34). This indicated that the z-score increased along with the increase of fetal fraction, with the possibility of true positivity increasing. However, no similar correlation was found in the T13 true positive group. It is possible that there are relatively few cases of T13, and the positive predictive value is influenced by the incidence of T13 itself. In addition, the z-score of chromosome 18 and z-score of chromosome 13 in the negative group were negatively and positively correlated with fetal fraction, respectively (*r *= − 0.0276, *P *< 0.0001; *r *= 0.09256, *P *< 0.0001). The results was similar with previous research. One study found that there is a strong positive correlation between z-scores and fetal fraction in pregnant women with T21, T18, and T13 fetuses and a negative correlation was observed in T18-negative samples (35). Thus, the effect of fetal fraction should be taken into consideration when identifying chromosome aneuploidy by z-score. As fetal fraction is an essential parameter in NIPS, the effect of fetal trisomy on fetal fraction should be investigated further.

The results of this study showed that it is reasonable to carry out NIPS according to the current international and Chinese national norms. The fetal fraction was within the acceptable range, and gestational age, maternal age, and BMI showed definitive characteristics and had a distinct trend with fetal fraction. This large-scale study not only verifies the results of previous studies, but also provided different perspectives from previous studies. This is of great significance for genetic counseling and further supports the effective use of NIPS. However, this study had some limitations. Only the most important factors were included in this study, other factors that might affect fetal fraction were not included in the analysis, such as serum pregnancy-associated plasma protein and free β-subunit of human chorionic gonadotropin, which will be further analyzed in future studies. In addition, confirmatory results of pregnant women with positive NIPS results who refused invasive prenatal diagnosis were not obtained. Therefore, the relationship between fetal fraction and maternal, fetal, and experimental factors requires further research.

## Conclusions

A retrospective analysis was carried out to discuss the fetal fraction of a large sample size in the research. Our study indicated that fetal fraction is affected by various factors. Fetal fraction increased with increasing gestational age, and the increasing rate was different at different stages of gestation. The fetal fraction tended to decrease with increasing maternal age and BMI. A variety of factors can influence fetal fraction and subsequently the accuracy of NIPS. Moreover, the z-scores of chromosomes increased along with the increase of fetal fraction, with the possibility of true positivity increasing. Therefore, genetic counseling before and after NIPS is crucial. Genetic counselors should understand the advantages and limitations before performing NIPS, especially considering the factors that may affect fetal fraction. Genetic counselors should also know how to interpret NIPS results when they are obtained. In these ways, the benefits of using NIPS can be maximized, which is important for the wider and more rational use of NIPS.

## Data Availability

The original contributions presented in the study are included in the article/Supplementary Material, further inquiries can be directed to the corresponding author/s.
